# Prof. Ford

**Published:** 1854-06

**Authors:** 


### PROF. FORD.

This gentlemen who has been absent to the Eastern cities, has returned again to his home in good health and much gratified with his tour. He visited all the Eastern cities and their schools as well as the Hospital. The attentions bestowed upon him at these different places were most gratifying to himself and complimentary to the school of which he was the representative.

An additional cause of gratification arose from the solicitude expressed by the profession in the East, for the welfare of our institution, having heard of our success during the past winter.

Dr. Ford secured additional appliances for teaching, and made arrangements for more in the future.

